# *Brucella abortus* Ornithine Lipids Are Dispensable Outer Membrane Components Devoid of a Marked Pathogen-Associated Molecular Pattern

**DOI:** 10.1371/journal.pone.0016030

**Published:** 2011-01-07

**Authors:** Leyre Palacios-Chaves, Raquel Conde-Álvarez, Yolanda Gil-Ramírez, Amaia Zúñiga-Ripa, Elías Barquero-Calvo, Carlos Chacón-Díaz, Esteban Chaves-Olarte, Vilma Arce-Gorvel, Jean-Pierre Gorvel, Edgardo Moreno, María-Jesús de Miguel, María-Jesús Grilló, Ignacio Moriyón, Maite Iriarte

**Affiliations:** 1 Departamento de Microbiología y Parasitología, Universidad de Navarra, Pamplona, Spain; 2 Focal Area Infection Biology, Biozentrum of the University of Basel, Basel, Switzerland; 3 Centro de Investigación en Enfermedades Tropicales, Facultad de Microbiología, Universidad de Costa Rica, San José, Costa Rica; 4 Programa de Investigación en Enfermedades Tropicales, Escuela de Medicina Veterinaria, Universidad Nacional, Heredia, Costa Rica; 5 Centre d'Immunologie de Marseille-Luminy, Aix Marseille Université, Faculté de Sciences de Luminy, Marseille, INSERM U631, CNRS UMR6102, Marseille, France; 6 Instituto Clodomiro Picado, Facultad de Microbiología, Universidad de Costa Rica, San Pedro, Costa Rica; 7 Centro de Investigación y Tecnología Agroalimentaria (CITA), Unidad de Sanidad Animal, Gobierno de Aragón, Zaragoza, Spain; 8 Instituto de Agrobiotecnología, CSIC-UPNA-Gobierno de Navarra, Pamplona, Spain; University of California Merced, United States of America

## Abstract

The brucellae are *α-Proteobacteria* facultative intracellular parasites that cause an important zoonosis. These bacteria escape early detection by innate immunity, an ability associated to the absence of marked pathogen-associated molecular patterns in the cell envelope lipopolysaccharide, lipoproteins and flagellin. We show here that, in contrast to the outer membrane ornithine lipids (OL) of other Gram negative bacteria, *Brucella abortus* OL lack a marked pathogen-associated molecular pattern activity. We identified two OL genes (*olsB* and *olsA*) and by generating the corresponding mutants found that *olsB* deficient *B. abortus* did not synthesize OL or their lyso-OL precursors. Liposomes constructed with *B. abortus* OL did not trigger IL-6 or TNF-α release by macrophages whereas those constructed with *Bordetella pertussis* OL and the *olsB* mutant lipids as carriers were highly active. The OL deficiency in the *olsB* mutant did not promote proinflammatory responses or generated attenuation in mice. In addition, OL deficiency did not increase sensitivity to polymyxins, normal serum or complement consumption, or alter the permeability to antibiotics and dyes. Taken together, these observations indicate that OL have become dispensable in the extant brucellae and are consistent within the trend observed in *α-Proteobacteria* animal pathogens to reduce and eventually eliminate the envelope components susceptible of recognition by innate immunity.

## Introduction

The members of the genus *Brucella* are α-2 *Proteobacteria* that cause brucellosis, an important disease affecting livestock and wild life as well as human beings. These bacteria trigger only low proinflammatory responses in the initial stages of infection and, accordingly, they follow a stealthy behavior that allows them to reach sheltered intracellular niches before effective immunity activation. The outer membranes (OM) of brucellae are of critical importance in this strategy. Whereas most gram-negative have OM molecules bearing the pathogen-associated molecular patterns (PAMP) recognized by innate immunity, at least the *Brucella* OM lipopolysaccharide (LPS), lipoproteins and flagellin display a reduced PAMP [1], [2]. Moreover, smooth (S) brucellae such as *B. abortus* and *B. melitensis* have OMs that are unusually resistant to the disrupting action of bactericidal peptides and complement. Thus, periplasmic and internal PAMP-bearing molecules like peptidoglycan or DNA are not readily accessible to pathogen recognition receptors [1], [3]–[8]. The *Brucella* LPS is clearly implicated in these properties and there is evidence that other lipid molecules also contribute. *Brucella* OMs contain large amounts of phosphatidylcholine (PC) and blockage of the synthesis of PC with the subsequent replacement by phosphatidylethanolamine (PE) generates attenuation [9], [10]. Ornithine lipids (OLs) are present in relatively large amounts in *Brucella* [11] and, although they have interesting properties in other bacteria, have not been investigated. It has been reported that *Pseudomonas fluorescens* grown under conditions that increase OL content becomes more resistant to the polycationic lipopeptide polymyxin B indicating a connection between these amino lipids and the resistance to bactericidal peptides [12], [13]. Moreover, OLs of *Bordetella pertussis*, *Flavobacterium meningosepticum* and *Achromobacter xylosoxidans* display antagonistic effects on LPS endotoxicity as well as proinflammatory and inflammatory activity [14]–[18]. Such an activity in *Brucella* OL would be in apparent contradiction with the furtive behavior of these bacteria with respect to innate immunity. Therefore, it was of interest to know whether *Brucella* OLs play a role in the OM stability and resistance to polycations and whether they display a biological activity different from that of other OLs, including the evasion of pathogen recognition receptors.

## Results

### OLs are OM components of *B. abortus*

**Figure 1:**
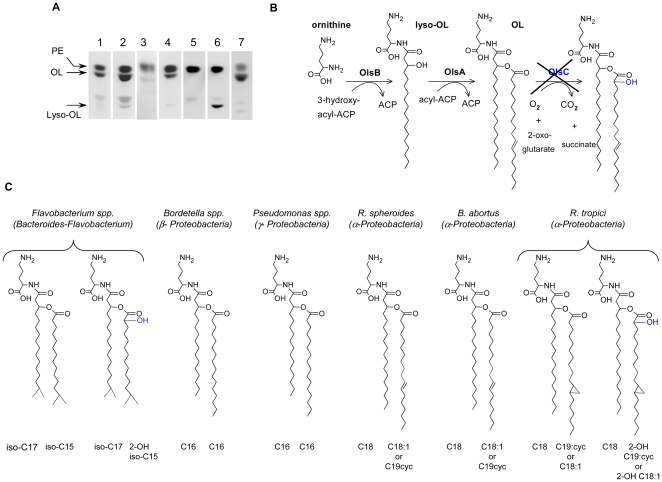
*Brucella abortus* OLs are OM components synthesized in a two step pathway. (A). Thin layer chromatography analysis of total free-lipid extracts of: (1), *BAB*-parental cells grown in tryptic soy broth; (2), OM fragments of *BAB*-parental; (3), *BAB*-parental crude LPS; (4) *BAB*-parental grown in minimal medium; (5), *BABΔolsB* cells; (6), *BABΔolsA* cells; and (7), *BABΔolsB* complemented with pLPI6. (B), proposed OL synthesis pathway [adapted from [87]]. The identities of *Brucella* OL acyl chains are from reference [88] and the genetic evidence. (C), proposed structures of OL of bacteria of various phylogenetic groups.

To determine the cellular localization of OLs, we first examined the free-lipids in virulent S *B. abortus* 2308 Nal^R^ grown in tryptic soy broth to the stationary phase, in the OM fragments released spontaneously during growth [19] and in non-delipidated *B. abortus* LPS [20]. Thin-layer chromatography of the corresponding chloroform∶methanol∶water extracts [21] confirmed the presence of OLs in *B. abortus* and showed their enrichment in the OM fragments (Figure 1 A), thus demonstrating that they are OM components. Although in amounts lower than PE, OLs were also detected in non-delipidated *B. abortus* LPS suggesting an association in the OM (Figure 1 A). The levels of OLs did not change when the bacteria were cultured in tryptic soy broth or in *Brucella* Gerhardt's minimal medium (lactate-glutamate-glycerol, mineral salts, vitamins) [22] (Figure 1 A).

### *B. abortus* OLs are synthesized through a two step pathway

We searched the *B. abortus* 2308 genome for orthologs of the genes involved in OL synthesis in other α-2 *Proteobacteria*. In *Sinorhizobium meliloti*, OlsB acylates the ornithine α amino group with C18:0(3-OH) and OlsA generates the acyloxyacyl group by esterification with C18:0 (Figure 1 B) [23], [24]. In *Rhizobium tropici*, an additional gene (*olsC*) codes for an oxygenase that generates a 2-hydroxy substitution on the ester-linked acyl group [25] (Figure 1 B). We found that ORF BAB1_0147 (annotated as encoding a hypothetical protein) codes for a protein with 55% identity and 66% similarity with OlsB, and that the product of ORF BAB1_2153 (annotated as a phospholipid-glycerol acyltransferase) has 45% identity and 61% similarity with OlsA. Moreover, we located similar ORFs in the genomes of *B. melitensis* 16M and *B. suis* 1330 (http://www.ncbi.nlm.nih.gov/genomes/lproks.cgi). ORF BAB1_0147 (henceforth *BABolsB*) maps as an isolated gene. BAB1_2153 (henceforth *BABolsA*) is in a possible operon formed by at least BAB1_2151 (putative glycoprotease), BAB1_2152 (putative acetyltransferase) and probably by BAB1_2154 (hypothetical protein; the stop codon of BAB1_2152 and the start codon of BAB1_2153 overlap). Both BABOlsA and BABOlsB contain a sequence [H73 (X)4 D78 and H76 (X)4 D81, respectively] that could correspond to the consensus motif [H(X)4D] of glycerolipid acyltransferases [26]. Moreover, BABOlsA contains two regions, NHTS (amino acids 72–75) and AEGTT (amino acids 143–147), closely resembling the NHQS and PEGTR motifs highly conserved in lysophosphatidic acid acyltransferases [27]. We also found a DNA sequence with homology to *olsC*. However, the possible BAB*olsC* carried a frame shift in the same position in all accessible *B. abortus*, *B. melitensis* and *B. suis* genomes so that it corresponds to two ORF (annotated as BMEI0464 and BMEI0465 in *B. melitensis* 16M). Amino acids 1 to 155 of BMEI0464 have a 87% identity (92% homology) with amino acids 31 to 185 of *S. meliloti* OlsC, and amino acids 1 to 94 of BMEI0465 are 87% identical (91% homology) to the 187 to 280 stretch of OlsC [28]. Most of the consensus of the OslC-LpxO family of proteins is in BMEI0464 but at the very end of the protein and truncated in the last four amino acids, including the last isoleucine conserved in all OlsC homologues [29]. All these characteristics strongly suggest that the structure of *Brucella* OLs is similar to those described previously for *Rhodospseudomonas sphaeroides* (Figure 1C).

Using the above-described evidence, we constructed internal in-frame deletion mutants of the virulent *B. abortus* 2308 Nal^R^ strain (henceforth BAB-parental) devoid of the consensus motifs following a PCR overlap strategy [30] (Table S1). For *BABolsB*, we removed amino acids 40 to 229, which resulted in a truncated protein of 107 amino acids (mutant *BABΔolsB*). The deletion in *BABolsA* (mutant *BABΔolsA*) eliminated amino acids 48 to 245 and resulted in a truncated protein of 69 amino acids. Figure 1 A shows that *BABΔolsA* lacked OLs but synthesized a new ninhydrin-positive component corresponding to the lyso-ornithine lipid (lyso-OL) precursor [31]. In contrast, the only ninhydrin-positive lipid generated by mutant *BABΔolsB* was PE. When we complemented mutant *BABΔolsB* with plasmid pLPI-6 (carrying *BABolsB*; Table S1), OL synthesis was restored (Figure 1 A). Similarly, *BABΔolsA* complemented with plasmid pYLI-1 (carrying *BABolsA*) was able to produce OLs (not shown). These results are consistent with a two step pathway in which BABOlsB and BABOlsA act consecutively and where deletion of the former abrogates OL and lyso-OL synthesis (Figure 1 B).

Characterization of mutants *BABΔolsA* and *BABΔolsB* showed no change in colonial morphology, or in catalase, oxidase, and urease activities. They were S according to lysis by *B. abortus* S-specific phages, agglutination with anti-A and anti-M monospecific sera, crystal violet exclusion and acriflavine agglutination test.

### OLs are not required for *Brucella* OM resistance to bactericidal peptides and complement

**Figure 2:**
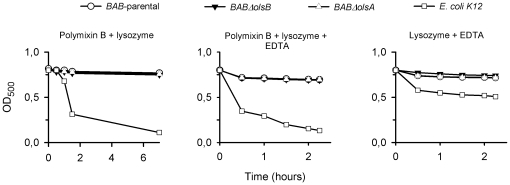
*B. abortus* OL-deficient mutants are not sensitive to polymyxin B, lysozyme or EDTA. Late exponential phase bacterial suspensions in HEPES (pH 7.5) were exposed to combinations of polymyxin B (100 units/ml), lysozyme (50 µg/ml) and EDTA (5 mM) and bacterial lysis followed turbidimetrically.

Due to the OL abundance in *Brucella* and their zwitterionic nature, it has been proposed that they play a relevant role in the stabilization of negative charges of LPS and, therefore, in the stability of the OM [32]. To test this, we first examined the sensitivity to bactericidal peptides. We found no significant differences in the minimal inhibitory concentrations of polymyxin B and colistin on *BAB*-parental, *BABΔolsA* and *BABΔolsB* strains. Since bactericidal peptides are also OM permeabilizing agents, we probed the mutants with polymyxin B plus lysozyme under hypotonic conditions in comparison with *Escherichia coli*. This treatment was effective in killing *E. coli* but had no action on mutants *BABΔolsB* or *BABΔolsA* or on *BAB*-parental (Figure 2). Moreover, EDTA alone or combined with polymyxin B did not promote lysozyme uptake, proving that divalent cations were not taking over the hypothetical role of OLs in OM stabilization (Figure 2). Finally, sensitivity to a set of antibiotics (penicillin, doxycycline, clarithromycin, erythromycin and rifampicin) that penetrate the OM by hydrophilic or hydrophobic pathways, or of dyes like thionine blue, fuchsine and safranin remained unchanged (not shown). All these results indicate that OLs are neither necessary to stabilize the OM of *B. abortus* against bactericidal peptides nor influence its permeability.

**Figure 3:**
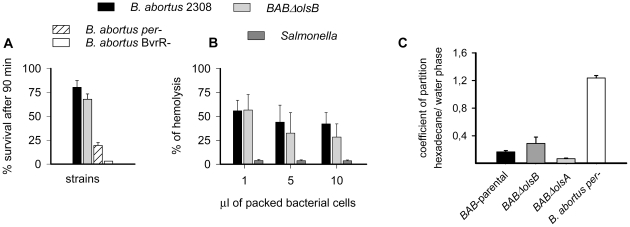
*B. abortus* OL-deficient mutants do not show increased sensitivity to normal serum, complement activation activity or altered surface hydrophobicity. (A), survival of *BAB-*parental and *BABΔolsB* after 90 min of incubation in non-immune serum (*B. abortus per- and B. abortus BvrR-* are mutants defective in the LPS O-polysaccharide or with an altered OM, respectively, that are sensitive to non-immune serum); (B), packed bacteria were incubated with normal rabbit serum and the complement remaining measured as the hemolytic activity using an erythrocyte-antibody system; (C), partition coefficients of of *BAB-*parental, *BABΔolsB*, *B. abortus per-* in hexadecane and water. Data are the mean ± standard error of triplicate measurements.

*B. abortus* mutants altered in PC synthesis, with a truncated LPS or an upset OM protein profile are sensitive to killing by non-immune serum [33]–[35]. However, OL deficiency did not have a similar effect because we observed only a small and not significant (p>0.05) increase in serum sensitivity in mutant *BABΔolsB* (Figure 3 A). Although small differences in antibody-independent complement activation were suggested when large amounts of bacteria were used (Figure 3 B), the differences were not statistically significant (p>0.05). Finally, we also tested the surface hydrophobicity of the mutants and the exposure of major OM proteins. The partition coefficient of the wild type *BAB*-parental and the *BABΔolsB* mutant in water∶hexadecane was similar and widely different from that of an O-polysaccharide deficient *B. abortus per* mutant used as a reference (Figure 3 C). Similarly, Western-blot analysis of extracts of wild type *BAB-*parental and *BABΔolsB* cell envelopes did not show differences in the reactivity of Omp 1, Omp2b, Omp31b, Omp25, Omp19, Omp16, and Omp10 (not shown).

### The absence of OLs does not influence proinflammatory responses to *B. abortus* or the ability to multiply intracellularly and generate chronic infections

**Figure 4:**
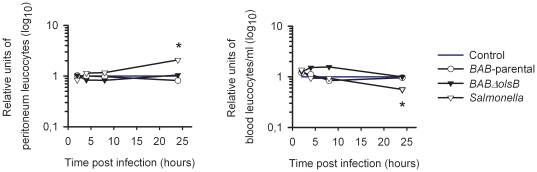
*B. abortus* OL-deficient mutants do not trigger increased blood or peritoneal leukocyte responses during the early stages of infection in mice. Mice were intraperitoneally injected with 10^6^ CFU of *BAB-*parental or *BABΔolsB* or with 10^5^ CFU of *S. typhimurium*, total leukocyte numbers determined at the indicated periods and values normalized with respect to the values in mice inoculated with PBS. Asterisks indicate significant differences between *S. typhimurium* and the control (no significant differences were observed for the *B. abortus* strains).

**Figure 5:**
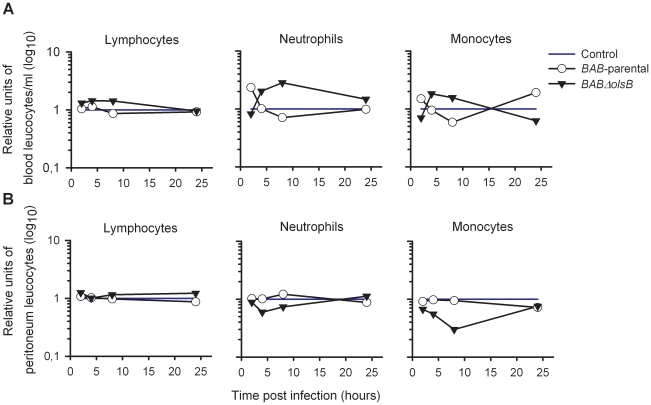
The OL-deficiency does alter not alter the profiles of lymphocytes, neutrophils and monocytes during the early stages of *B. abortus* infection in mice. Lymphocyte, neutrophil and monocyte number is (A) blood and (B) peritoneum of mice intraperitoneally injected with 10^6^ CFU of *BAB-*parental or *BABΔolsB* (values normalized with respect to the values in mice inoculated with PBS).

**Figure 6:**
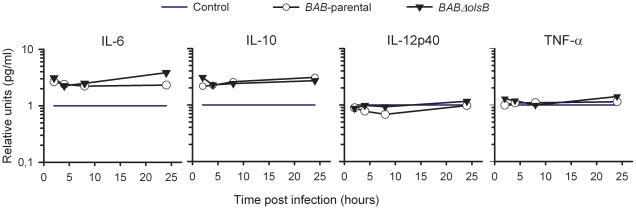
The OL-deficiency does not alter cytokine responses to *B. abortus* during the early stages of infection in mice. IL-6, IL-10, IL-12p40 and TNF-α levels in the blood of mice intraperitoneally injected with 10^6^ CFU of *BAB-*parental or *BABΔolsB* (values normalized with respect to the values in mice inoculated with PBS).

Since *B. abortus* induces a negligible proinflammatory response in mice at early times of infection [1], we examined whether the lack of OLs could alter this property by examining several markers. First, we tested the generation of fibrin D-dimer that would result from the activation of the blood clothing cascade caused by endotoxic microorganisms. For this, we infected Swiss Webster mice 10^6^ CFU/mouse of *BAB-parental* or mutant *BABΔolsB* (controls received 0.1 ml of 100 mM phosphate buffered saline (pH 7.2) [PBS]) and examined the serum 24 h later. For *BAB-*parental, the results confirmed previous reports [1] on the absence of any increase in fibrin D-dimers above the positive threshold (0.5 µg/ml). Similarly, infection with the OL-deficient mutant did not have a significant effect on fibrin D-dimer generation. Then, we determined the number of leukocytes in the peritoneal fluid and blood of mice injected intraperitoneally with 10^6^ CFU of *BAB-parental* or *BABΔolsB*, 10^5^ CFU of *S. typhimurium* or 0.1 ml of PBS. Whereas the latter induced a leukocyte peritoneal recruitment and a progressive reduction in blood leukocyte numbers at later times, these linked effects were not observed in *BAB*-parental or *BABΔolsB* mutant infected mice (Figure 4). Similarly, lymphocyte, neutrophil and monocyte numbers in blood and peritoneal fluid did not reveal significant differences in *Brucella* infected mice (Figure 5). We also measured TNF-α, IL-6, IL-10 and IL-12p40 in the blood of the same animals, and found that the normalized levels of these cytokines were similar in *BAB-*parental and *BABΔolsB* infections (Figure 6). These levels were similar to and much lower than those reported for *B. abortus* 2308 and *S. typhimurium*, respectively [1].

**Figure 7:**
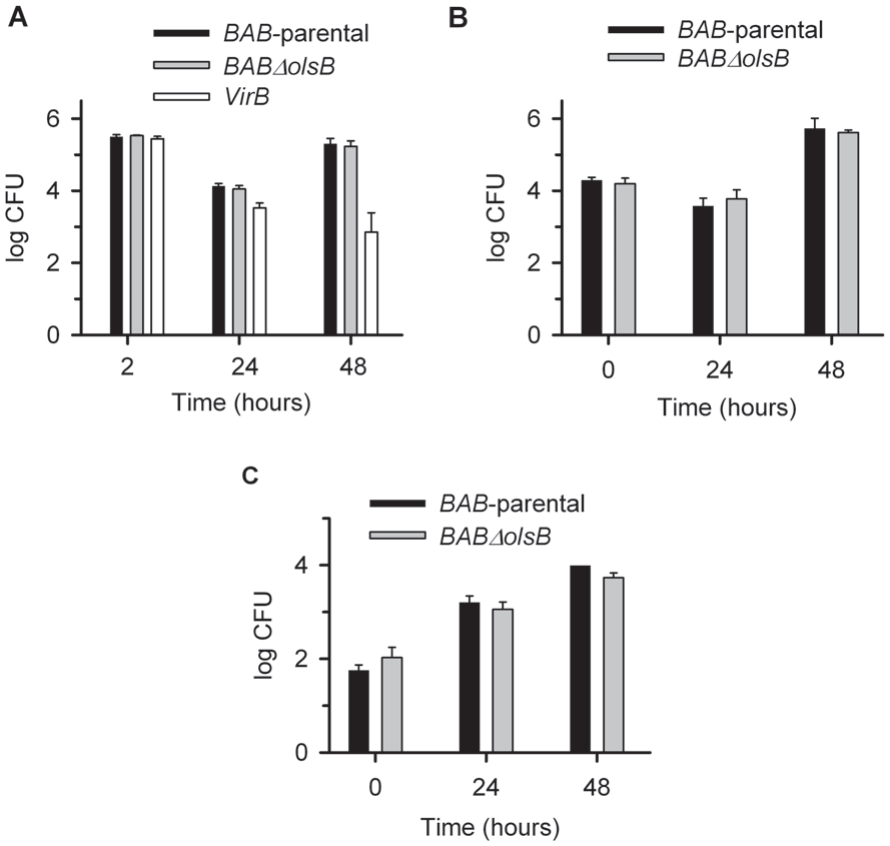
The OL-deficiency does not alter the ability of *B. abortus* to multiply in mouse cells. (A), bone-marrow derived macrophages; (B), RAW 264.7 macrophages; (C), Hela cells. Values are the mean ± standard error of triplicate infections, and the results shown are representative of three independent experiments.

To complement the above-described studies, we tested the ability of the *BABΔolsB* OL-deficient mutant to multiply in cells and to generate chronic infections. First, we infected bone marrow derived macrophages, RAW 264.7 macrophages and HeLa cells and monitored the intracellular survival of bacteria [36]. Figure 7 shows that the *BABΔolsB* mutant retained the ability to multiply intracellularly in all these cells. Then, we inoculated BALB/c mice intraperitoneally with 5×10^4^ CFU/mouse of *BABΔolsB* or of *BAB-*parental. Two, 6, and 12 weeks later, the bacteria in the spleens were counted, the identity of the isolates confirmed by PCR, and the spleen weights recorded. The results showed that the parental were practically identical throughout the experiment (average log_10_ CFU ± SD values for *BABΔolsB* and *BAB-*parental, respectively, were: week 2, 6.09±0.24 and 6.20±0.11; week 6, 6.65±0.19 and 6.69±0.32; and week 12: 5.85±0.48 and 5.80±0.46). Similarly, there were no splenomegaly differences (0.35±0.05 and 0.40±0.04; 0.58±0.06 and 0.57±0.12; 0.46±0.09 and 0.43±0.12).

Consistent with the experiments *in vitro*, these observations suggest that the absence of OLs does not affect the OM stability *in vivo* and, accordingly, that OLs seem not to hamper the release of PAMP-bearing molecules and the establishment of chronic infections. Moreover, since *BABΔolsB* did not promote a lower or higher proinflammatory response than *BAB-*parental, the results suggest that these lipids are neither detected by the pathogen recognition receptors nor antagonists in the recognition of other OM molecules during brucellosis.

### *B. abortus* OL do not stimulate cytokine secretion in murine macrophages

**Figure 8:**
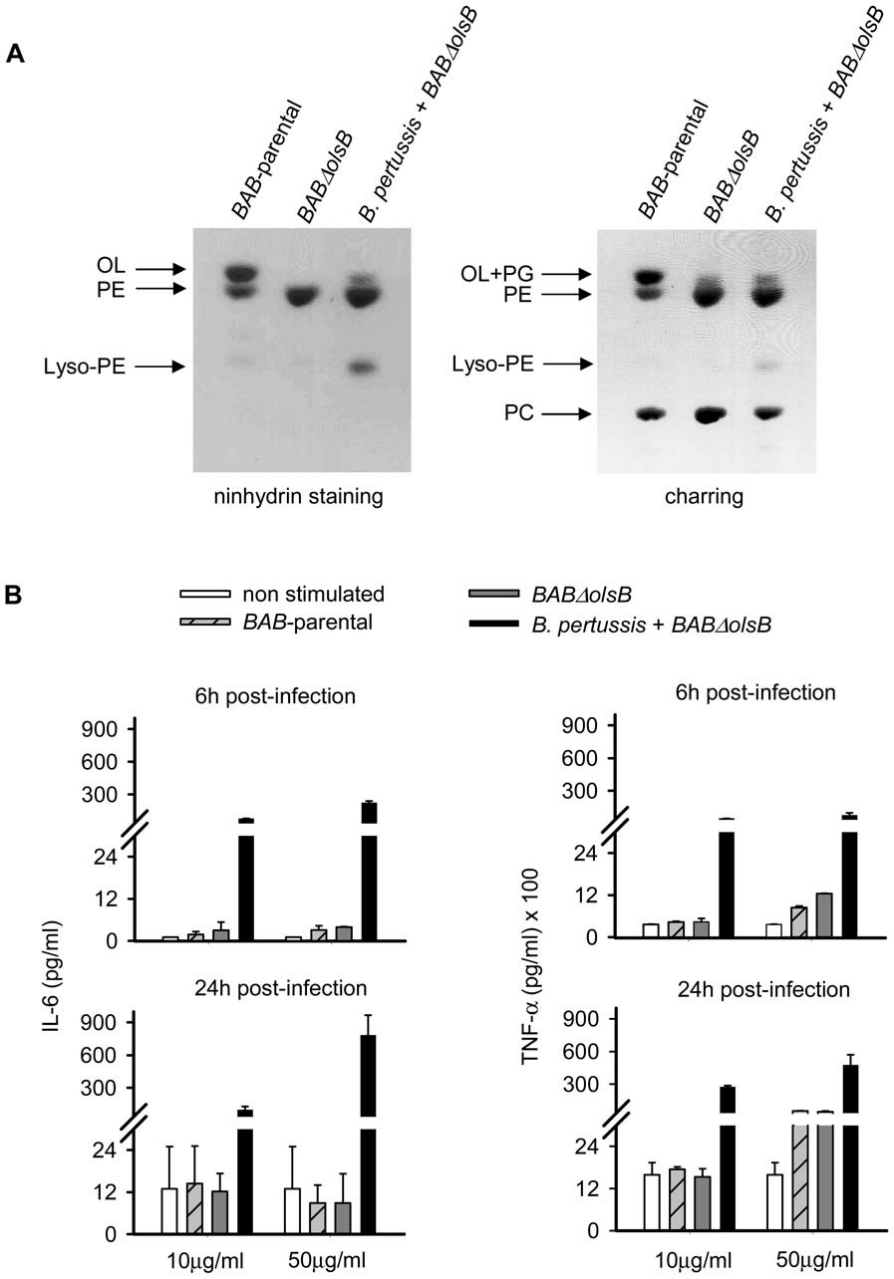
*B. abortus* OL do not stimulate TNF-α and IL-6 release in murine macrophages. Liposomes were made with the free lipid fraction of *BAB*-parental, *BABΔolsB*, or a mixture of *B. pertussis* and *BABΔolsB* free lipids. (A), lipid composition of liposomes (aminolipids: ninhydrin staining; total lipids: sulfuric acid charring); (B), IL-6 and TNF-α in the supernatants of RAW 264.7 macrophages after stimulation for 6 and 24 h with the indicated liposomes.

To test whether *Brucella* OLs carry a PAMP, we used the *BAB-*parental and *BABΔolsB* lipids, since the presence of PC in *Brucella* allows obtaining stable liposomes. As a positive control, we extracted the lipids of *B. pertussis* (containing OL but not PC) and generated *B. pertussis* OL-liposomes using the OL-free lipids of *BABΔolsB* as carriers. After verification of the liposome composition (Figure 8 A), we stimulated RAW 264.7 macrophages. While the *B. pertussis*-*BABΔolsB* liposomes notably stimulated TNF-α and IL-6 secretion, the *BAB-*parental or *BABΔolsB* liposomes were inactive (Figure 8 B). These results clearly show that *Brucella* OL, in contrast to those of *B. pertussis*, do not bear a marked PAMP. In addition, they demonstrate that *Brucella* phospholipids do not inhibit PAMP recognition.

## Discussion

The OM of most gram negative bacteria hinders the penetration of harmful molecules, and the LPS plays a key role in this important property. The LPS inner sections (core oligosaccharide and lipid A) are rich in acidic sugars and orthophosphate, and these negatively charged groups bind divalent cations and polyamines that bridge the LPS molecules and hamper the partition of hydrophobic permeants into the OM [37], [38]. However, this supramolecular arrangement makes OM sensitive to divalent cation chelators like EDTA and to bactericidal peptides. Moreover, this set of properties is connected to the PAMP of a variety of OM molecules, primarily the LPS. Interestingly, *Brucella* OM is comparatively permeable to hydrophobic compounds and resistant to those agents. At least in part, these properties are accounted for by a low number of negatively charged groups which are limited to two 2-keto-3-doxyoctulosonic acid residues and the lipid A phosphates [39]–[43]. Moreover, it was proposed that the *Brucella* OLs could shield those negatively charged groups by virtue of their positively charged amino groups, as postulated for *P. fluorescens* [44]. Indeed, the results of this and previous works in other bacteria [45]–[47] support that *Brucella* OLs are in fact N-(acyloxyacyl)- ornithine OM structural elements with a free amino group that should be positively charged at neutral and acidic pH. Such an OL role would represent an advantage for a pathogen because the hydrophobic anchorage should make OLs more resistant than divalent cations to displacement by bactericidal peptides and proteins. However, since OL deficiency did not increase the sensitivity of *Brucella* cells to polymyxins or the permeability to lysozyme (a cationic peptide), this hypothesis was disproved. Furthermore, any possible defect not detected by the *in vitro* methods seems not to be relevant *in vivo*, at least in cells and mice. One possibility is that OLs are in the inner leaflet of the OM and, therefore, not in contact with the polar moiety of the LPS. In fact, our results suggest that PE (also with a free amino group) is the more important LPS associated-lipid in *B. abortus*.

The interactions of OLs with innate immunity have been analyzed in some γ and β *Proteobacteria*. The OLs from *B. pertussis* and *A. xylosoxidans* stimulate IL-1, TNF-α and prostaglandin E2 synthesis in macrophages [14], [48]–[50]. Moreover, *F. meningosepticum* OLs are mitogenic for B-lymphocytes and exhibit adjuvant activity in C3H/HeJ mice, suggesting that receptors other than TLR4 are involved in OL recognition [14], [51], [52]. Consistent with these observations, OLs of *B. pertussis* presented in *BABΔolsB* liposomes triggered cytokine release. Therefore, the fact that this effect was significantly lower when similar liposomes carried *B. abortus* OLs demonstrates that innate immunity fails to efficiently recognize these *Brucella* amino lipids. This is a well-known property of *Brucella* LPS and other *Brucella* putative PAMP bearing molecules such as flagella and lipoproteins and, therefore, our results extend this ability to another OM element [1], [53]. It is known that the acyl groups in LPS, other glycolipids and synthetic aminolipids or lipopeptides modulate the inflammatory activity [54]–[56] and, indeed, the acyl chains of *Brucella* OL differ from those of other bacteria in length and, in some cases, in the presence of a hydroxyl group (Figure 1 C) [57]–[61]. In some bacteria, OL have ester-linked fatty acyl groups with a hydroxyl group at the 2-position and this hydroxyl group may affect the biological activity [62]. However, this hydroxylation is a post-synthesis modification that requires OlsC, which is inactive in *Brucella* according to our genomic analysis. All these data strongly suggest that the reduction of *Brucella* OL PAMP is connected to the acyl chains, longer in α-*Proteobacteria* than in other phylogenetic groups (Figure 1 C). Indeed, these results add more weight to the hypothesis that the hydrophobic moieties of *Brucella* OM elements are critical in avoiding recognition of this pathogen by innate immunity [1], [8], [63].

Here, we also presented evidence that *Brucella* OLs are dispensable elements. Taking into account that *Brucella* OLs are quantitatively important lipids, it is surprising that they are dispensable. However, the α-*Proteobacteria* show a marked tendency to live in tight interactions with eukaryotic cells [64] and there is evidence that this ability is associated to a reduction in envelope molecule PAMPs. *Bartonella* possess a low endotoxic LPS, a reduced number of OM lipoproteins and flagellins that are not recognized by TLR5 [65]–[67]. Similarly, *Rickettsia* carry a non canonical LPS and a reduced number of lipoproteins [68], and *Wolbachia* do not posses genes to synthesize LPS, flagella or fimbriae, have a low number of lipoproteins and an unusual peptidoglycan [69]. Finally, the genomes of *Ehrlichia* and *Anaplasma* contain a low number of lipoprotein genes and do not have the genetic machinery to synthesize LPS, peptidoglycan or flagellin [70], [71]. Accordingly, we hypothesize that free-living *Brucella* ancestors carried OLs that were useful OM structural elements in their environment but lost their importance once the major OM target of immunity (i.e. the LPS) became adapted to the host. This adaptation was marked by the modification in LPS PAMP connected features (i.e., acyl chains and charged groups) and had as a result that LPS no longer needed stabilizing agents in the OM. Within this perspective, the degeneracy of the *olsC* homologue is in keeping with the hypothesis that *B. abortus* OL represent dispensable ancestral structures that could be eventually eliminated during the evolutionary process.

## Materials and Methods

### Ethics Statement

All animals were handled and sacrificed according to the approval and guidelines established by the “Comité Institucional para el Cuido y Uso de los Animales” of the Universidad de Costa Rica, and in agreement with the corresponding law “Ley de Bienestar de los Animales No 7451” of Costa Rica (http://www.micit.go.cr/index.php/docman/doc_details/101-ley-no-7451-ley-de-bienestar-de-los-animales.html).

BALB/c mice (Charles River, Elbeuf, France) were accommodated in the animal building of the CITA of Aragón (ID registration number ES-502970012005) in cages with water and food ad libitum and under biosafety containment conditions, for 2 weeks before the start and all along the experiment. The animal handling and procedures were in accordance with the current European legislation (directive 86/609/EEC) supervised by the Animal Welfare Committee of the institution (protocol number R102/2007).

### Bacterial strain and growth conditions

Bacteria were grown in tryptic soy broth or agar either plain or supplemented with kanamycin (Km) at 50 µg/ml, or/and nalidixic (Nal) at 25 µg/ml, or/and gentamicin (Gm) at 20 µg/ml, or/and chloramphenicol at 20 µg/ml (all from Sigma), or/and 5% sucrose. Where indicated, the defined medium of Gerhardt [72] was used. All strains were stored in skim milk at −80°C. The origin of the *B. abortus* and *S. typhimurium* strains is described in previous works [1], [73], [74]. *B. pertussis* is a clinical isolate kindly provided by G. Martínez de Tejada.

### Bacteriological procedures, antibiotic sensitivity and cell surface characterization

The mutants were characterized according to standard *Brucella* typing procedures [75]: colonial morphology after 3 days of incubation at 37°C, crystal violet exclusion, catalase, oxidase, urease, acriflavine agglutination, sensitivity to Tb, Wb, Iz and R/C phages, agglutination with anti-A and anti-M monospecific sera, CO_2_ and serum dependence, and susceptibility to thionine blue, fuchsine, and safranin. Moreover, the minimal inhibitory concentrations of polymyxin B, colistin, penicillin, doxycycline, clarithromycin, erythromycin and rifampicin were determined in Müller-Hinton medium by standard procedures.

Surface hydrophobicity was analyzed as described by Kupfer and Zusman [76]. Bacteria were grown in tryptic soy broth until stationary phase, incubated in 0.5% sodium azide at 37°C overnight, collected by centrifugation (7000×*g*, 10 min, 4°C), washed twice with a solution of K_2_HPO_4_.3H_2_0 97mM, KH_2_PO_4_ 53mM, urea 21mM, MgSO_4_.7H_2_O 0.8mM and resuspended to an OD_470_ = 1.0. A volume of 1.5 ml of this bacterial suspension was mixed with 0.5ml of n-hexadecane and incubated during 10 min at 37°C. After shaking for 40 seconds, the tubes were settled to allow phases separation to occur. The partition was calculated as (1−OD_470_ of water phase)/OD_470_ of the water phase. Western-blot analysis with monoclonal antibodies to the major Omps was carried out as described before [77].

### DNA manipulations, construction of mutants and complementation

Plasmid and chromosomal DNA were extracted with Qiaprep spin Miniprep (Qiagen GmbH, Hilden, Germany), and Ultraclean Microbial DNA Isolation kit (Mo Bio Laboratories) respectively. When needed, DNA was purified from agarose gels using Qiack Gel extraction kit (Qiagen). DNA sequencing analysis was performed by the Servicio de Secuenciación de DNA del Centro de Investigación Médica Aplicada (Navarra, Spain). Primers were synthesized by Sigma-Genosys Ltd.. Searches for DNA and protein homologies were carried out using the NCBI (National Center for Biotechnology Information (http://www.ncbi.nlm.nih.gov) and the EMBL-European Bioinformatics Institute server (http://www.ebi.ac.UK/ebi_home.html). In addition, sequence data were obtained from The Institute for Genomic Research at http://www.tigr.org. Genomic sequences of *B. melitensis* 16M, *B. abortus* and *B. suis* were analyzed using the database of the L'Unité de Recherche en Biologie Moléculaire (URBM, Namur, Belgium) (http://www.serine.urbm.fundp.ac.be/~seqbruce/GENOMES/Brucella_melitensis).

For constructing the *BAB*Δ*olsB* mutant, oligonucleotides *olsB*-F1 (5′ -CTTCTGTCATCGTCGCGTAG- 3′) and *olsB*-R2 (5′-GATGCGTCCCAGAATGATG-3′) were used to amplify a 270-bp fragment including codons 1 to 39 of the *olsB* ORF, as well as 153 bp upstream of the *olsB* first putative start codon, and oligonucleotides *olsB*-F3 (5′-CATCATTCTGGGACGCATCCCAAAGGAAGCGATCAACAA-3′) and *olsB*-R4 (5′- TTAAAACCGGAACCGCTCTA- 3′) were used to amplify a 294-bp fragment including codons 230 to 297 of the *olsB* ORF and 87-bp downstream of the *olsB* stop codon. Both fragments were ligated by overlapping PCR using oligonucleotides *olsB*-F1 and *olsB*-R4 for amplification, and the complementary regions between *olsB*-R2 and *olsB*-F3 for overlapping. The resulting fragment, containing the *olsB* deletion allele, was cloned into pCR2.1 (Invitrogen), to generate plasmid pIRI-2, sequenced to ensure the maintenance of the reading frame, and subcloned into the BamHI and the XhoI sites of the suicide plasmid pJQ200KS. The mutator plasmid (pLRI-7) was introduced in *BAB-*parental by conjugation [78]. Integration of the suicide vector was selected by Nal and Gm resistance, and the excision (generating the mutant strain by allelic exchange) was selected by Nal and sucrose resistance and Gm sensitivity. The resulting colonies were screened by PCR with primers *olsB*-F1 and *olsB*-R4 which amplify a fragment of 564 bp in the mutant and a fragment of 1134 bp in the parental strain. The mutation resulted in the loss of the consensus amino acids responsible for the enzymatic activity.

The *BAB*Δ*olsA* mutant was constructed using primers *olsA*-F1 (5′- GATTGCGCAGGATACCATCT -3′) and *olsA*-R2 (5′-AACAATGCGGTGGAAGAAAT-3′) to amplify a 498- bp fragment including codons 1 to 47 of the *olsA* ORF, as well as 357-bp upstream of the *olsA* start codon and primers *olsA*-F3 (5′ATTTCTTCCACCGCATTGTTACGATGGAAAATCGGGTGAG- 3′) and *olsA*-R4 (5′-CAGCGCGGAATAGAGTTTTT- 3′) to amplify a 372-bp including codons 246 to 268 of the *olsA* ORF and 303 bp downstream of the *olsA* stop codon. Both fragments were ligated by overlapping PCR using primers *olsA*-F1 and *olsA*-R4 and the fragment obtained, containing the deletion allele, was cloned into pCR2.1 to generate pYLI-2, sequenced to confirm that the reading frame had been maintained, and subcloned in pJQ200KS to produce the mutator plasmid pYLI-3. This plasmid was introduced in *BAB-*parental and the deletion mutant generated by allelic exchange was selected by Nal and sucrose resistance and Gm sensitivity and by PCR using oligonucleotides *olsA*-F1 and *olsA*-R4 which amplify a fragment of 870 bp in the deletion strain and a fragment of 1464 bp in the parental strain. The mutation generated results in the loss of 73.8% of the *olsA* ORF.

For complementation, plasmids carrying *olsB* and *olsA* were constructed using the Gateway cloning Technology (Invitrogen). Gene *olsB* was amplified from *BAB-*parental using primers *olsB*- F13 (5′ GGGGACAAGTTTGTACAAAAAAGCAGGCTTCATGACAGCACTGCTTGGAATGG 3′) and gene *olsB*- R14 (5′ GGGGACCACTTTGTACAAGAAAGCTGGGTC CTAGACAAAGCGGTTTGCTTC 3′), that contain the *attB* sequences (underlined), and cloned into vector pDONR221 (Invitrogen) to generate pLPI-5. The ORF was subsequently cloned in pRH001 [79], able to multiply in *Brucella*, to produce the complementation plasmid pLPI-6. Since the sequence of *olsA* from *B. abortus* and *B. melitensis* is 99% identical, the clone carrying *olsA* was extracted from the *B. melitensis* ORFEOMA [80] and the ORF subcloned into plasmid pRH001 [81] to produce plasmid pYLI-1. To complement the *olsB* mutation, plasmid pLPI-6 was introduced into the *BABΔolsB* mutant by mating with *E. coli* S17 λpir and the conjugants harboring this plasmid (designated as *BABΔolsB* pLPI-6) were selected by plating the mating mixture onto tryptic soy agar-Nal-Km plates. The *olsA* mutation was complemented following the same protocol by introducing plasmid pYLI-1 into the *BABΔolsA* mutant.

### Lipid analysis

Total lipids were extracted as described by Bligh and Dyer [82], and analyzed on silica gel 60 high-performance thin layer chromatography plates (Merck Chemicals), the plates were pre-washed by solvent migration with chloroform–methanol-water (140∶60∶10, vol/vol), dried thoroughly. Then samples were applied and chromatography performed in the same mixture of solvents [83]. Plates were developed with 0.2% ninhydrin in acetone and heating at 120°C for 5 min or by charring with 15% sulfuric acid in ethanol at 180°C. L-β,γ-dipalmitoyl-α cephalin (Sigma-Aldrich) was used as a standard.

### Sensitivity to non immune serum and complement consumption

Exponentially growing bacteria were adjusted to 10^4^ CFU/ml and mixed with fresh sheep normal serum (45 µl of cells plus 90 µl of serum per well) in microtiter type plates in duplicate. After incubation for 90 min at 37°C with gentle stirring, brain heart infusion broth (200 µl/well) was added, mixed and 100 µl aliquots plated out by triplicate. The results were expressed as the % survival with respect to the CFU in the inocula. Complement consumption was estimated as the reduction of the hemolytic activity of rabbit serum complement incubated with live bacteria, and *S. typhimurium* SL1344 was used as a positive control [1].

### Infections and bacterial counts in cells

Bone marrow cells were isolated from femurs of 6–10-week-old C57Bl/6 female mice and differentiated into macrophages [84]. Infections were performed at a multiplicity of infection of 50∶1 by centrifuging bacteria onto macrophages at 400 *g* for 10 min at 4°C, followed by a15 min incubation at 37°C under a 5% CO_2_ atmosphere. Macrophages were extensively washed with Dulbeccos's modified Eagle's medium to remove extracellular bacteria and incubated for an additional 90 min in medium supplemented with 50 µg/ml gentamicin to kill extracellular bacteria. Thereafter, the antibiotic concentration was decreased to 10 µg/ml. At each time point, samples were washed three times with 100 mM PBS before processing. Alternatively, murine RAW 264.7 macrophages (Raw 264.7; American Type Culture Collection No. TIB-71) or Hela cells (American Type Culture Collection No. CCL-2) were used in a similar protocol. To monitor *Brucella* intracellular survival, infected cells were lysed with 0.1% (vol/vol) Triton X-100 in H_2_O after PBS washing and serial dilutions of lysates were rapidly plated onto TSB agar plates to enumerate CFU. The attenuated *B. abortus virB* mutant (Table S1) was used as a control.

### OL stimulation of macrophages

For macrophage stimulation, OL were included in liposomes. Total free-lipid extracts of *BAB-*parental, *BABΔolsB* or a 1∶1 mixture of *B. pertussis and BABΔolsB* free-lipids were evaporated under a nitrogen stream, resuspended thoroughly in 250 µl of 10mM HEPES to a final concentration of 5 mg/ml, and the composition verified by thin-layer chromatography. Phase contrast microscopy and fluorescein entrapment controls demonstrated that *Brucella* total lipids formed liposomes that were stable for at least 24 h without addition of exogenous lipids. The murine RAW 264.7 macrophages used in these experiments were grown at 37°C under 5% CO_2_ in Dulbecco's medium supplemented with 10% fetal bovine serum, 2.5% sodium bicarbonate, 1% glutamine (all from Gibco), penicillin (100 units/ml) and streptomycin (100 µg/ml). Macrophages (5×10^5^ cells) were treated with 20 µg/mL or 100 µg/mL of liposomes of *BAB*-parental, *BAB*ΔolsB or *B. pertussis-BAB*ΔolsB in 10% fetal calf serum-Dulbecco's Modified Eagle's Medium. After 90 min of incubation, fresh 10% fetal calf serum- Dulbeccos's modified Eagle's medium was added to obtain a final concentration of 10 µg/ml and 50 µg/ml of liposomes, and the amount of TNF-α and IL-6 in the supernatants was assessed by ELISA (eBioscience) after 6 and 24 h.

### Proinflammatory responses in mice

Swiss CD1 mice of 18 to 20 g were used. Fibrin D- dimers were determined from the plasma of mice 24 h after intraperitoneal infection with 0.1 ml of bacteria (1×10^6^ UFC/mouse) in pyrogen-free PBS (pH 7.2). Fibrin D-dimers were assessed by the semiquantitative D-Di test® latex agglutination assay (Diagnostica Stago). The levels of IL-6, IL-10, IL-12p40 and TNF-α were estimated by ELISA (eBioscience) in the sera of mice (n = 5) infected intraperitoneally with 10^6^ CFU of *BABΔolsB* or *BAB-*parental. For leukocyte counts, mice were intraperitoneally infected with 1×10^6^ CFU of *BABΔolsB* or *BAB*-parental or 10^5^ CFU of *S. typhimurium* SL1344 in pyrogen-free sterile PBS (pH 7.2), or with pyrogen-free sterile PBS as a control. Blood was collected from the retrorbital plexus at different time points in tubes with 2mg/ml of EDTA. Then, 5 ml of ice cold PBS were injected in the peritoneal cavity, and 3.0 to 4.5 ml of fluids were collected with a syringe. After centrifugation, the peritoneal cells were resuspended in 0.2 ml of PBS, and total leukocytes, neutrophils, monocytes and lymphocytes were counted. The number of cells recruited in the peritoneum was corrected according to the volume of fluid collected from each animal [1].

### Virulence and splenomegaly in mice

Groups of 30 mice (seven-week-old female BALB/c mice [Charles River, Elbeuf, France]) were inoculated intraperitoneally with 5×10^4^ CFU/mouse of *BABΔols*B or *BAB-*parental in 0.1 mL of PBS, and the spleen weights and number of viable bacteria in spleens were determined in five mice at 2, 6, and 12 weeks post-inoculation. Experimental procedures (i.e. preparation and administration of inocula, retrospective assessment of the exact inoculating doses, and determination of the number of CFU/spleen) were performed as described previously [85]. The identity of the spleen isolates was confirmed by PCR amplification at each selected post-inoculation point time. Spleen weights were expressed as the mean and SD (n = 5) of grams/spleen and infections as mean ± SD (n = 5) of log_10_ CFU/spleen at each selected post-inoculation point-time, previous logarithmic normalization of individual data. Statistical comparisons between means were performed by ANOVA and the Fisher's Protected Least Significant Differences test [86].

## Supporting Information

Table S1 **Bacterial strains and plasmids.**
(DOC)
