# Supplementary material for: Brucella abortus Ornithine Lipids Are Dispensable Outer Membrane Components Devoid of a Marked Pathogen-Associated Molecular Pattern
Source: PLoS One. 2011 Jan 7;6(1):e16030. doi: 10.1371/journal.pone.0016030 (PMC3017556; doi:10.1371/journal.pone.0016030)
Supplement: Table S1 — Bacterial strains and plasmids. (DOC) [file pone.0016030.s001.doc]

Table S1. Bacterial strains and plasmids.

| **Strain / plasmid** | **Characteristics** | **Reference /Source** |
| --- | --- | --- |
| ***Brucella abortus*** |  |  |
| *B. abortus* 2308 Nal^R^  *(BAB*- parental) | Wild type, virulent, biotype 1, smooth LPS, Nal^R^ spontaneous mutant of strain 2308 | [1] |
| *BABΔolsB* | 2308 Nal^R^ *olsB* _Δ40-229_ | This work |
| *BABΔolsA* | 2308 Nal^R^ *olsA* _Δ48-245_ | This work |
| *BABΔolsB* pLPI-6 | 2308 Nal^R^ *olsB* _Δ40-229_ harboring plasmid pLPI-6 encoding *BABolsB* encoding *BABolsB* | This work |
| *BABΔolsA* pYLI-1 | 2308 Nal^R^ *olsA* _Δ48-245_ harboring plasmid pYLI-1 encoding *BABolsA* | This work |
| 9.49 per | 2308 Nal^R^ *per*::Tn5; rough-LPS | [2] |
| *virB* | 2308 Nal^R^ mutant deleted of *virB10* | [3] |
| 65.21 | 2308 Nal^R^ *bvrR*::Tn5, smooth LPS | [4] |
| ***E. coli*** |  |  |
| S17λpir | Mating strain with plasmid RP4 inserted into the chromosome | [5]  [6] |
| TOP10 F’ | F - *lacI*q Tn *10* (Tetr) *mcrA* Δ(*mrr-hsdRMS-mcrBC*) 80*lac*ZΔM15 Δ*lacX74 recA1alaD139* Δ (*ara-leu*)*7697 galU galK rpsL endA1 nupG* | Invitrogen |
| **Plasmids** |  |  |
| pCR2.1 | Cloning vector, Km^R^ | Invitrogen |
| pDONR221 | Cloning vector, Km^R^ | Invitrogen |
| pJQ200KS | Sac suicide vector, Gm^R^ | [7] |
| pRH001 | Derivative of pMR10 Km^R^; Cm^R^ | [8] |
| pIRI-2 | 564-bp of *B. abortus* chromosomal DNA containing the *BABolsB* deletion allele, generated by PCR and cloned into pCR2.1 | This work |
| pLRI-7 | *BamHI*-*Xho*l fragment from pIRI-2 cloned into the corresponding sites of pJQ200KS | This work |
| pYLI-2 | 870-bp of *B. abortus* chromosomal DNA containing the *BABolsA* deletion allele, generated by PCR and cloned into pCR2.1 | This work |
| pYLI-3 | *Xba*l-*BamHI* fragment from pYLI-2 cloned into the corresponding sites of pJQ200KS | This work |
| pLPI-5 | *B. abortus* chromosomal DNA containing the complete *olsB* gene, with the *att*B sites*,* generated by PCR and cloned into pDONR221 | This work |
| pDONR201- BMEI1977 | *B. melitensis* chromosomal DNA containing the complete *olsA* gene, generated by PCR and cloned into pDONR201 (Invitrogen) | [9] |
| pLPI-6 | *att*L1- *att*L2 fragment of pLPI-5 cloned into the *att*R*1*- *att*R*2* sites of pRH001 | This work |
| pYLI-1 | *att*L1- *att*L2 fragment of pDONR201- BMEI1977 cloned into the *att*R1- *att*R2 sites of pRH001 | This work |

References

1. Sangari F, Aguero J (1991) Mutagenesis of *Brucella abortus*: comparative efficiency of three transposon delivery systems. Microb Pathog 11: 443-446.

2. Monreal D, Grilló MJ, González D, Marín CM, de Miguel MJ et al. (2003) Characterization of *Brucella abortus* O-polysaccharide and core lipopolysaccharide mutants and demonstration that a complete core is required for rough vaccines to be efficient against *Brucella abortus* and *Brucella ovis* in the mouse model. Infect Immun 71: 3261-3271.

3. Sieira R, Comerci DJ, Sánchez DO, Ugalde RA (2000) A homologue of an operon required for DNA transfer in *Agrobacterium* is required in *Brucella abortus* for virulence and intracellular multiplication. J Bacteriol 182: 4849-4855.

4. Sola-Landa A, Pizarro-Cerdá J, Grilló MJ, Moreno E, Moriyón I et al. (1998) A two-component regulatory system playing a critical role in plant pathogens and endosymbionts is present in *Brucella abortus* and controls cell invasion and virulence. Mol Microbiol 29: 125-138.

5. Simon LD, Randolph B, Irwin N, Binkowski G (1983) Stabilization of proteins by a bacteriophage T4 gene cloned in *Escherichia coli*. Proc Natl Acad Sci U S A 80: 2059-2062.

6. Miller VL, Mekalanos JJ (1988) A novel suicide vector and its use in construction of insertion mutations: osmoregulation of outer membrane proteins and virulence determinants in *Vibrio cholerae* requires *toxR*. J Bacteriol 170: 2575-2583.

7. Quandt J, Hynes MF (1993) Versatile suicide vectors which allow direct selection for gene replacement in gram-negative bacteria. Gene 127: 15-21.

8. Hallez R, Letesson JJ, Vandenhaute J, de Boelle X (2007) Gateway-based destination vectors for functional analyses of bacterial ORFeomes: Application to the min system in *Brucella abortus*. Appl Environ Microbiol 73: 1375-1379.

9. Dricot A, Rual JF, Lamesch P, Bertin N, Dupuy D et al. (2004) Generation of the *Brucella melitensis* ORFeome version 1.1. Genome Res 14: 2201-2206.
